# Can virtual events achieve co-benefits for climate, participation, and satisfaction? Comparative evidence from five international Agriculture, Nutrition and Health Academy Week conferences

**DOI:** 10.1016/S2542-5196(21)00355-7

**Published:** 2022-02-09

**Authors:** Joe Yates, Suneetha Kadiyala, Yuemeng Li, Sylvia Levy, Abel Endashaw, Hallie Perlick, Parke Wilde

**Affiliations:** a Faculty of Epidemiology and Population Health, London School of Hygiene & Tropical Medicine, London, UK; b Gerald J. and Dorothy R. Friedman School of Nutrition Science and Policy, Tufts University, Boston, MA, USA

## Abstract

The advancement of science and evidence-based solutions for planetary health increasingly require interdisciplinary and international learning and sharing. Yet aviation travel to academic conferences is carbon-intensive and expensive, thus perpetuating planetary health and equity challenges. Using data from five annual international Agriculture, Nutrition and Health Academy Week conferences from 2016 to 2020, we explore whether moving to virtual conferencing produced co-benefits for climate, participation, attendee interaction, and satisfaction. We report on: absolute number of attendees, proportion of attendees from countries of different income levels, number of participants at social events, aviation CO_2_ emissions, and overall ratings of the event by participants. Transitioning online resulted in large reductions in travel-related aviation CO_2_ emissions, alongside increased attendance—including among attendees from low-income and middle-income countries. This was achieved without a major change in the participant rating of the event. However, the online format resulted in lower participation in conference social events. The urgency of reducing CO_2_ emissions in pursuit of planetary health and improving equity in scientific exchange requires new modalities of academic conferencing. This study indicates that co-benefits can be achieved when transitioning online. Challenges exist for virtual events, such as emulating the intangible facets of in-person interactions, overcoming time-zone limitations, and digital divides.

## Introduction

The twin challenges of climate change and COVID-19 have intensified global interest in new methods of coordinating and communicating scientific research, particularly on critical topics such as planetary health. Responding to concerns about climate change, a rapidly growing literature has measured greenhouse gas emissions from academic and research conferencing and explored lower-carbon alternatives.^1^, ^2^, ^3^ Coupled with this are ongoing debates around efforts to decolonise global health^4^ and evidence on inequity in scientific symposia, where costs are high and participation from low-income and middle-income countries (LMICs) is disproportionately low.^5^ Financial barriers, social constraints, opportunity costs, and lack of accessibility hamper meaningful participation by traditionally under-represented groups, including early career researchers, women, researchers from low-income contexts, minority groups, and those with disabilities, among others.^6^ A large fraction of international conference emissions come from participant air travel. Before 2020, aviation transportation accounted for 3% of annual global CO_2_ emissions.^5^ An in-person global research conference can entail travel emissions of 1·3–1·8 tonnes of CO_2_ emissions per attendee,^7^ which exceeds two-thirds of the total annual per capita emissions for India (1·9 tonnes) and a substantial fraction of emissions even for the USA (16·6 tonnes) or the UK (5·6 tonnes).^8^, ^9^, ^10^ A study of the 2019 annual conference of the American Geophysical Union described alternatives with lower emissions: enhancing virtual presence would reduce travel-related emissions by 39%; shifting to a fully virtual format could reduce them by 99%.^11^

While maintaining networks and sharing work has always been important for scientific communities, planetary health and food systems research agendas are inherently multidisciplinary. Thus, forming international collaborations and learning and sharing across disciplines and geographies is both urgent and critical. A high value is therefore placed on broad-based engagement and locating conferences appropriately to alleviate existing inequities of opportunity among under-represented groups such as early career researchers and those in LMICs, for whom costs can be prohibitive.^12^ However, debates continue about the extent to which overseas travel can improve academic success.^13^ The emphasis placed on face-to-face meetings to foster scientific collaboration might have constrained past efforts to make international conferences more sustainable and equitable.^14^

**Key messages**

• International collaboration is critical to addressing planetary health challenges
• Academic conferences can be expensive, emissions-intensive, and inequitable
• The COVID-19 pandemic has accelerated transitions to online or hybrid conference formats
• One such transition reduced travel-related aviation CO_2_ emissions and increased attendance across all country income levels without affecting overall participant rating
• However, uptake of opportunities to network socially declined
• Hybrid events and improved technology must rise to these challenges

Since the beginning of 2020, the COVID-19 crisis has delivered an unprecedented shock to global research conferencing. For organisations helping to coordinate international research, innovations in online conferencing previously contemplated for slow adoption were instead planned and executed within mere months. This Viewpoint reports on such a transition for the Agriculture, Nutrition and Health (ANH) Academy Week conference, the flagship annual event for a global network of over 5000 researchers, practitioners, and policy makers from over 100 countries, working at the intersections of agriculture–food systems, nutrition, and health. The ANH Academy Week serves as a platform for learning and sharing, creating space and opportunities for interaction across disciplines, geographies, academic seniority, and sectors. Since its launch in 2016, it has rotated between Africa and Asia in order to lower barriers to participation (especially among scientists in these regions), to support leadership of host institutions around these topics, and to foster equitable interdisciplinary global partnerships. The ANH Academy operated a travel bursary scheme to support over 130 speakers from low-income settings. In 2016, there was no registration fee; from 2017 to 2019, a nominal contribution ranging from US$50 to $75 was charged predominantly for participants from high-income countries (free for host countries and waived for most other participants on request), while the online event in 2020 was free of charge. Between 2016 and 2019, the annual ANH Academy Week conference was held in Addis Ababa, Ethiopia; Kathmandu, Nepal; Accra, Ghana; and Hyderabad, India respectively, with the 2020 event initially planned for Lilongwe, Malawi (appendix p 1). Since 2018, efforts to make the event more sustainable included livestreaming and plastic-free conference materials, food, and beverages. The ANH Academy planned to offset the travel-associated emissions of the originally planned in-person 2020 event with financial support of sustainable forestry and livelihood programmes in Malawi, recognising that offsetting is no substitute for actual emissions reduction.

The sudden transition to an online ANH Academy Week conference in July, 2020 due to COVID-19, after four in-person events, offered a unique opportunity to analyse data from this natural experiment across a 5-year period and by conference formats.

We investigated the following questions: could a virtual conference increase absolute number of participants, its geographical reach, and participation from LMICs? Could it do so without compromising participant satisfaction? What are the savings in aviation-related CO_2_ emissions when moving from in-person to a fully online conference? What challenges do virtual conferences continue to present?

## Methods

We focused on three outcome domains, primarily using data routinely collected by the ANH Academy for its annual ANH Academy Week conferences: (1) participation: attendance and geographical spread, and participant interaction at ANH Academy Week social events; (2) climate: aviation-related CO_2_; and (3) participant satisfaction.

### Participation

#### Attendance and geographical spread

We counted as participants those who actually attended the event (physically signed in to an in-person event or attended a live Zoom session for the online event), as opposed to those who simply registered or registered and viewed the conference sessions and material posted online. We report the absolute number of attendees for each year.

For the reach of the conference, we present data on the number of different countries from which participants attended the conference each year. These data are captured routinely during registration under the field “Your location”. We categorised participants’ country location by the World Bank's fiscal year 2021 country income categories: low ($1035 gross national income [GNI] per capita or less), lower middle ($1035–4045 GNI per capita), upper middle ($4046–12 535 GNI per capita) and high ($12 536 or more GNI per capita).^15^ For the four in-person conferences (2016–2019), country location was reported for all participants. For the virtual conference (2020), information on country was missing for 54 participants (6%); hence, adjusted results assigned them proportionally to the four country categories (unadjusted results are provided in the appendix [p 1]). In the World Bank fiscal year 2021 classification, Ethiopia (2016) was a low-income country, while the other three in-person sites were in lower-middle-income countries: Nepal (2017), Ghana (2018), and India (2019). We used a constant classification year, because the alternative of using current classification for the conference year would make it more difficult to distinguish changes in attendance patterns from changes in country classification. The only host country to switch income categories during the study period was Nepal, which changed from low income to lower middle income in fiscal year 2021.

#### Participant interaction at social events

ANH Academy Week conferences present opportunities for informal participant interaction. For each of the in-person events (2016–2019) this entailed three centrally organised social receptions open to all participants—two on-site and one off-site—as well as an enabling environment for participants to organise their own social or community-of-practice events. For 2020, this offering was emulated, but adapted to cater for the online format through a range of “social hangouts” (3–4 per day) that were trialled alongside adverts for participants to organise their own socials that the organisers would facilitate.

We present the cumulative number of participants who took part in social events, normalised per 100 participants per year. Participants might have taken part in multiple social opportunities, so this measure is a function both of the number of social events and the uptake per social event. For in-person events, participants were manually counted by organisers; for online events, participant data were generated automatically by Zoom software. Although this approach does not provide information on the specific duration of participation of each participant in each of the social events, or richness of informal interactions—and is not a pure measure of uptake—it does provide a high-level indication of the amount of opportunity to socially interact.

### Climate

Aviation travel CO_2_ emissions from fuel burn were estimated using the Atmosfair online calculator.^16^ In addition, non-CO_2_ effects related to water vapour and contrail formation can be up to twice as large as the CO_2_ effects, but these non-CO_2_ effects are shorter-lasting and more difficult to measure, so, to be conservative, they are excluded in our estimates.^17^ For attendees located in a conference host country, we assumed intra-country ground travel (not counted in air travel emissions), except for Hyderabad, India (2019) due to large intra-country distances (1600 km from Delhi, for example). For attendees from outside the host country, we assumed air travel from the largest airport, except for Benin and Togo, which were less than 400 km from Accra, Ghana (2018). We recognise that some intra-Africa travel might have included an intercontinental leg; however, we do not have flight path data for each attendee across four in-person conferences. Because we included only the shortest flight path and included only CO_2_ emissions, our approach conservatively underestimates the full impact on global climate.

Based on previous evidence that aviation is responsible for most conference emissions,^7^, ^11^ we did not estimate non-aviation conference emissions. Food and beverage intake, and embedded costs for electronic equipment (computers, monitors, and telephones), might not differ for in-person and virtual conferences, because attendees consume food and beverages and use their own computers and cell phones with both formats. Marginal emissions from electric transmission (video streaming) were found by Klöwer and colleagues to be responsible for less than 1% of conference CO_2_ emissions.^11^

### Participant satisfaction

Each year, all ANH Academy Week conference participants were emailed a link to complete an anonymous online feedback survey soon after the event. The surveys asked participants to respond to the following question: “Please rate the [year] ANH Academy Week” using a five-point Likert scale question with 1 being “Poor” and 5 being “Excellent”. For 2016, participants were asked to rate each of the individual sessions on this five-point scale with 5 being labelled as “Outstanding”; for this year only, we present data aggregated from individual sessions to enable comparison between events. The survey questions and full distribution of results are reported in the appendix (pp 1–4). The survey did not elicit information on respondent characteristics. The original motivation for collecting feedback was to improve the event. The potential use of the data to compare different conference formats only became apparent after the onset of the COVID-19 pandemic and its challenges in 2020.

We also report on some additional observations gained from delivering in-person and online ANH Academy Week conferences that would be useful in understanding how to continue working towards optimising co-benefits of scientific exchange in an equitable and sustainable way.

### Ethics approval

Ethics approval was granted from the London School of Hygiene & Tropical Medicine Interventions Research Ethics Committee (date approved Feb 22, 2021; reference number 22656).

## Results

### Participation

#### Attendance and geographical spread

**Figure 1:**
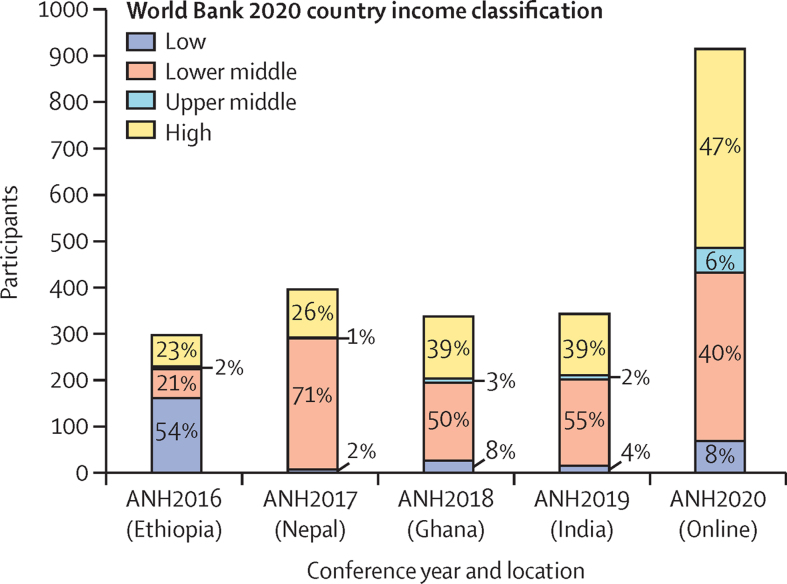
Number of ANH Academy Week participants by country income category

For the four in-person conference years (2016–19), the number of ANH Academy Week conference attendees ranged from 297 to 397 individuals (figure 1). For the virtual event year (2020), total attendance was much higher, 914 individuals.

Most participants (58–75%) were from low-income and lower-middle-income countries for the in-person conference years (2016–19). The proportion of participants from low-income countries was highest in 2016, the year that Ethiopia was the host country. The proportion of participants from lower-middle-income countries was highest in 2017, the year that Nepal was the host country. Nepal, which had 226 participants in 2017 (57% of all participants), would have been classified as low-income by the World Bank classification that year.

The absolute number of participants from low-income and lower-middle-income countries increased for the virtual conference year (2020), but their proportion was lower (47%) than previous in-person event years. The number of participants from upper-middle-income and high-income countries in 2020 (481) represented a four-fold increase (416%) compared with the average number of attendees from these country categories between 2016 and 2019 (figure 1).

Attendees of the in-person ANH Academy Week conferences came from 37 unique countries in 2016 (Ethiopia), 23 in 2017 (Nepal), 36 in 2018 (Ghana), and 37 in 2019 (India). By contrast, the online event in 2020 attracted participants located in 71 different country locations. The average number of unique LMICs that were represented between 2016 and 2019 was 23, while the online conference in 2020 attracted participants from 46 unique LMICs.

#### Participant interaction at social events

**Figure 2:**
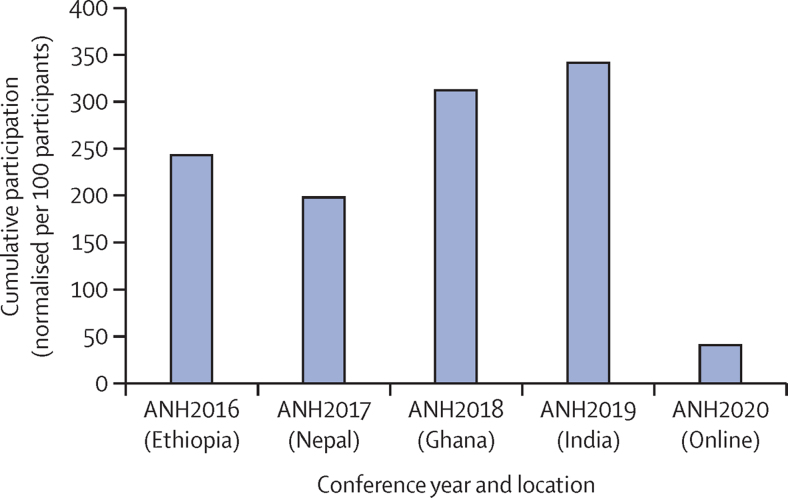
Participation in social events Cumulative number of participants who participated in social events offered at ANH Academy Weeks, normalised per 100 participants.

Between 2016 and 2019, the in-person events saw a steady increase in the cumulative number of people participating in social events, ranging from 541 in Ethiopia in 2016, to 802 in India in 2019. For the online event in 2020 this dropped by over 50% to 391 (figure 2). When normalised per 100 participants, participation in social opportunities offered by the ANH Academy Week decreased in 2020 to 14% of the average between 2016 and 2019.

### Climate

**Figure 3:**
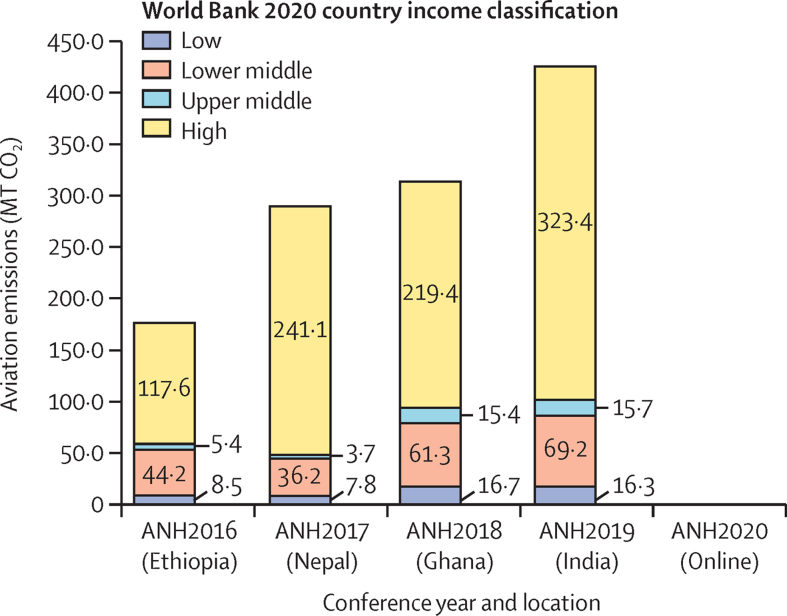
Total air travel CO_2_ emissions for ANH Academy Week conferences, by participant country income category MT CO_2_=metric tonnes of carbon dioxide.

Total CO_2_ emissions for air travel rose across the 4 years of in-person conferences (2016–19), due to both increases in the number of participants and per-participant emissions (figure 3). The overall per-participant CO_2_ emissions rose from 591 kg for Ethiopia (2016) to 1238 kg for India (2019).

Given the events’ intentional location in lower-middle-income countries, and the large number of participants attending from Europe and North America, most CO_2_ emissions for air travel were attributable to participants from high-income countries, which ranged from 67% to 83% of total CO_2_ emissions for air travel. Similarly, per-participant CO_2_ emissions for air travel were much higher for the high-income countries than overall, with emissions of more than 1600 kg per participant for the two destinations in Africa (Ethiopia in 2016 and Ghana in 2018) and more than 2300 kg per participant for the two destinations in Asia (Nepal in 2017 and India in 2019).

### Participant satisfaction

**Figure 4:**
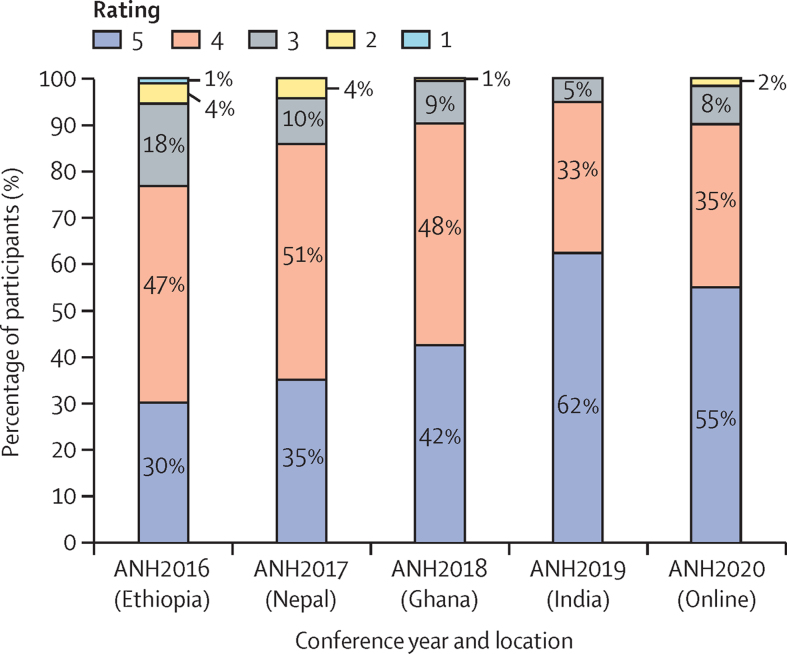
Participant rating of ANH Academy Weeks Ratings are 1–5 on a Likert Scale, with 1 being “Poor” and 5 being “Excellent” (or “Outstanding”, in ANH2016). Expressed as percentage of survey respondents per rating.

The survey response rate ranged from 30% to 45% for the in-person conferences—within the average range of email surveys^18^—and was 20% for the virtual conference (2020). With these response rates, non-response bias is possible. As noted earlier, the feedback surveys did not capture respondent demographic characteristics; therefore, the extent of non-response bias is unknown. Participant ratings of the event improved across the four in-person conferences, with the second-highest (4) or highest (5) rating rising from 76% of responses in Ethiopia (2016) to 95% in India (2019), with 90% for the virtual (2020) conference. The ratings across the Likert Scale over the 5 years is presented in figure 4 and the appendix (p 1).

### Additional observations

First, programming the online conference that aims to reach a global audience was restricted by time zones, more so than is the case with an in-person event. This resulted in a smaller programme and more parallel sessions, which somewhat limited the interdisciplinary nature of the ANH Academy—which started out in 2016 with a strict policy of plenary sessions only to facilitate cross-disciplinary learning.

**Figure 5:**
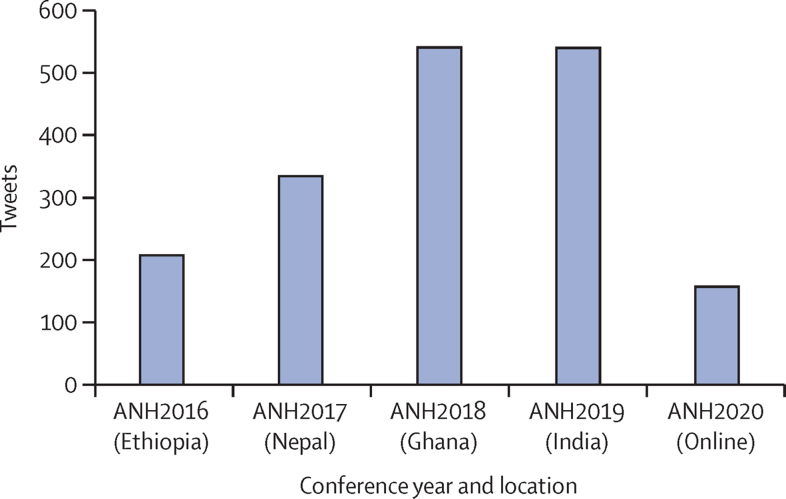
Twitter engagement Total tweets, retweets, and replies using the official ANH Academy Week hashtag each year, normalised per 100 participants.

Second, measuring engagement in scientific events can be attempted through different metrics, from assessing website analytics and session attrition to counting question and answer activity and monitoring chatboxes. Although we did not capture data for the latter (and neither is such comparable data possible for in-person *vs* online events), we observed rich and intensive chatbox activity from early career researchers during online conference sessions in 2020. In the future, these data could be used effectively to understand the depth and extent of participant interaction. We did, however, capture Twitter activity. Modern conferences often designate specific hashtags to stimulate participant engagement and expand conversations to wider audiences.^19^, ^20^ The ANH Academy Week conference employed a consistent hashtag format of “#ANHYYYY” (eg, #ANH2016 for 2016). Using data from Vicinitas and Twitter Analytics, we present the total number of tweets, retweets, and replies that use the official conference hashtag each year. In the health sciences literature,^21^ social media engagement metrics have been developed at three hierarchical levels: low (Facebook likes and Twitter followers), medium (blog comments and Twitter retweets), and high (new content from the user). We acknowledge that this Twitter hashtag metric reflects a combination of medium and high levels of engagement. Twitter activity varied from year to year, with the virtual (2020) conference generating the second lowest absolute number of tweets with the official hashtag (404), two-thirds that of the highest in Nepal, 2017, with 606 tweets. Proportional to total participation (tweets per 100 participants) the virtual 2020 event had the least Twitter activity (156 tweets) compared with 2019 (540), 2018 (541), 2017 (334), and 2016 (206), where “tweets” include total tweets plus retweets plus replies with the official hashtag (figure 5).

Third, both in-person and online events are not without their technical frailties. For in-person ANH Academy Week conferences, we have observed issues with audio-visuals equipment, power, and visa and transport issues among others, while the online event in 2020 suffered from website overloads, bandwidth issues for participants, and complete whole-day internet outages in Ethiopia and Malawi.

## Discussion

The advancement of science for impact relies on international conferences for scientific exchange, learning and sharing emerging findings, and setting research agendas. Equitable participation requires efforts to improve opportunities and inclusion for diverse researchers from all parts of the world. Planetary health requires reducing emissions of greenhouse gases. When the COVID-19 crisis forced the 2020 ANH Academy Week conference, originally planned to be hosted in Malawi, to move online, a key question was whether a new virtual format could preserve and enhance these multiple objectives in an equitable and sustainable way.

Our results suggest that the online format offered strengths in reducing aviation greenhouse gas emissions while enhancing geographical reach and attendance of researchers from both high-income countries and LMICs, thus agreeing with recent similar evidence.^22^ For the in-person conferences, air travel emissions increased each year from 2016 to 2019, where a total of 425 tonnes of CO_2_ were produced for the conference in India. These emissions represented 1·2 tonnes of CO_2_ per participant (mean), which is more than three-fifths of the 1·9 tonnes a typical person in India emits during an entire year. For the 2020 online event, aviation-related CO_2_ was zero.

The 2020 event benefited from the reputation, momentum, and social capital built by four preceding in-person conferences. However, transitioning overnight to a virtual format, especially with the challenges brought by COVID-19 for both the organisers and the ANH Academy Week conference participants, had limitations for networking and interaction objectives. Accommodating all time zones of the world necessitated more parallel sessions than usual, which might have hampered interdisciplinary discussion and learning. While the 2020 online event provided some imaginative “social hangouts” and encouraged attendee-organised networking events, participation in opportunities for social interaction and Twitter activity were much higher in-person than online. This might have been due to competing demands that existed in people's home and work environments under COVID-19 lockdowns, differences in time zones, and “digital divides” in access to reliable internet connections and equipment.^23^ These issues present new challenges for online conferences that seek to leverage multiple co-benefits of learning and sharing in equitable and sustainable ways.^24^

The online format developed in short notice limited the ability of early career researchers to interact with senior researchers in an informal manner, which had been a strength of in-person ANH Academy Week conferences. However, virtual events can facilitate new types of participant interaction and lower accessibility barriers that might exist in the physical realm, for instance by democratising Q&A through chatboxes, using live captions and creating virtual spaces for poster discussions, and establishing social groups and platforms to connect researchers professionally for scientific exchange and collaborations.^6^, ^14^ Furthermore, although conference sessions at ANH Academy Weeks have always been recorded and posted online, the uptake was lower before 2020 when there may have been a change in attitude, culture, and competency to engage with online content. Virtual events can be programmed imaginatively using platforms that enable attendees to enjoy the entire conference, pause and rewind talks, and take in the science at their own pace.^18^ Looking forward, we anticipate that continued innovation will improve the effectiveness of online conferences for networking and social interaction, but in-person formats will retain their comparative advantage in this area.

Air travel remains a major barrier to participation for early career researchers from LMICs with fewer resources and for those with family responsibilities or disabilities. This is further compounded by registration fees (although not for ANH Academy Weeks) and visas, accommodation and subsistence costs, opportunity costs, as well as the time lost in transit.^6^ Although our data do not permit a definitive conclusion, the higher online attendance and the doubling of LMIC geographical reach might have been enabled by lower financial and opportunity costs. However, the participation from high-income countries grew proportionally faster than that from LMICs, which might have been due to weaker infrastructure, lower bandwidth and connectivity, and fewer IT resources per households in LMICs; this suggests that there might be continued barriers to participation for researchers from lower-income settings. Internet outages in Ethiopia and Malawi during the 2020 online event reflect the challenges that online and hybrid conferences must address proactively to realise the goal of equitable scientific exchange.

This study demonstrates that an international conference of this size and complexity can be transitioned to an online format with short notice, delivering large emissions reductions and increased attendance and geographical reach. Our results suggest that these co-benefits were realised largely without compromising the perceived quality of the event.

### Strengths and limitations

This study provides a novel opportunity to compare key metrics for climate impacts, equity of attendance, and participant satisfaction in substantially different conferencing formats. Although there is a rich and growing literature on aviation-related emissions from individual academic conferences,^2^, ^3^, ^11^ as well as participant evaluations of different conference formats^25^—or both combined^26^—a strength of this study is comparing these two formats, alongside data on geographical participation and interaction, using evidence from a well-established in-person conference and its online counterpart. That this transition happened overnight provides valuable insights into what is both possible and challenging in such unanticipated circumstances.

Given the nature of this natural experiment, it is not without limitations, which should be accounted for when interpreting results. Firstly, for each outcome domain, the available underlying data sources were designed for conference management rather than research. Given that the participant data was originally collected to improve the event, we did not seek comprehensive informed consent, which limits the analysis and reporting using the rich data we have. Linked to this, the lower response rate for 2020 participant feedback might be due in part to the constraints on people's time during COVID-19 lockdowns, and thus means these data should be treated with caution.

Despite the limitations of retrospectively harnessing data not originally planned for research purposes, their analysis at this unique historical moment in time and context facilitates the comparison of key outcomes that can support the further design of future conferencing models seeking to improve scientific exchange, equity, and sustainability.

## Conclusion

Using the example of the ANH Academy Week conference, we have shown that large air travel-related emissions savings can be gained by transitioning an international conference to an online format. Important challenges for virtual conferences remain in terms of emulating the intangible facets of in-person interactions, for which the pandemic has brought renewed appreciation. Yet, according to our findings, the environmental wins of the online 2020 conference were accompanied by increases in participation—both in absolute numbers and in the reach to unique countries. However, barriers to this participation could continue to exist, as evidenced by a less-than-expected proportional increase in attendance from low-income country researchers. Swinging the pendulum too far in the other direction (virtual conferences) before improving digital access in low-income countries runs the risk of further increasing the divide. These are challenges that future virtual conferences should explicitly plan to overcome. Indeed, not all equity issues around conference participation will necessarily be solved through virtual conferences, but the challenges presented by COVID-19 in 2020 have driven rapid changes in knowledge and attitudes surrounding such tools, technologies, and events, and have fast-tracked decades of slow-moving norms. The challenge now is to sustain this momentum, continue to innovate, and pilot new conference formats in order to bridge the gaps between in-person and online experiences. In this respect, although they might come with their own new challenges, there is potential for hybrid conference models to achieve the best of both worlds, so that the mutually reinforcing needs of planetary health and scientific collaboration might be realised in an equitable and sustainable way.

## Declaration of interests

JY, AE, SL, and SK are staff at the London School of Hygiene & Tropical Medicine (LSHTM) who organise the ANH Academy Week conferences. HP also works on the Innovative Methods and Metrics for Agriculture and Nutrition Actions (IMMANA) programme at Tufts University, of which the ANH Academy is a workstream. Together, they have an interest in improving the event each year. PW and YL are affiliated with the Multi-Site Conference Hosting Initiative (MULCH) programme, which has an interest in better understanding the potential for innovative conferencing formats and was a delivery partner in ANH2021.
