# Supplementary material for: Can virtual events achieve co-benefits for climate, participation, and satisfaction? Comparative evidence from five international Agriculture, Nutrition and Health Academy Week conferences
Source: Lancet Planet Health. 2022 Feb 9;6(2):e164–70. doi: 10.1016/S2542-5196(21)00355-7 (PMC8850368; doi:10.1016/S2542-5196(21)00355-7)
Supplement: Supplementary appendix [file mmc1.pdf]

THE LANCET
Planetary Health

Supplementary appendix

This appendix formed part of the original submission and has been peer reviewed. We post it as supplied by the authors.

Supplement to: Yates J, Kadiyala S, Li Y, et al. Can virtual events achieve co-benefits for climate, participation, and satisfaction? Comparative evidence from five international Agriculture, Nutrition and Health Academy Week conferences. *Lancet Planet Health* 2022; **6:** e164–70.

Can virtual events achieve co-benefits for climate, participation and satisfaction? Comparative evidence from five international Agriculture, Nutrition and Health Academy Week conferences

Joe Yates MSc[1], Suneetha Kadiyala Phd[1], Yuemeng Li IBDP[2], Sylvia Levy BSc[1], Abel Endashaw MSc[1], Hallie Perlick MPH[2], Parke Wilde PhD[2].

[1]London School of Hygiene & Tropical Medicine
[2]Tufts University Gerald J. and Dorothy R. Friedman School of Nutrition Science and Policy

Webappendix

Data tables and participant feedback surveys

A. Data table

SUMMARY DATA	ANH2016 (Ethiopia)	ANH2017 (Nepal)	ANH2018 (Ghana)	ANH2019 (India)	ANH2020 (Online)
**World Bank country classification (FY 2021)[1]**	Low income	Lower Middle Income	Lower Middle Income	Lower Middle Income	N/A
**Participation**					
**Total participants (across whole event)**	**297**	**396**	**337**	**343**	**914**
**Unique countries represented (by participant location)**	32	31	49	33	72
**Unique countries by FY 2021 World Bank classification of location (N, unadjusted)**					
Low income	8	2	9	5	11
Lower middle income	15	12	13	13	24
Upper middle income	3	1	5	5	11
High income	11	8	9	14	25
**Participants by FY 2021 World Bank classification of location (N, unadjusted)**					
Low income	161	7	27	15	65
Lower middle income	63	283	168	187	342
Upper middle income	6	2	9	8	50
High income	67	104	133	133	403
Unspecified	0	0	0	0	54
	**297**	**396**	**337**	**343**	**914**
**Participants by FY 2021 World Bank classification of home country (N, with proportional allocation of "unspecified")**					
Low	161	7	27	15	69
Lower middle	63	283	168	187	363
Upper middle	6	2	9	8	53
High	67	104	133	133	428
**Total**	**297**	**396**	**337**	**343**	**914**
Upper middle and high-income %	24.6%	26.8%	42.1%	41.1%	52.7%
**Cumulative participants at social events**					
Participants	541	594	827	923	399
Normalised (p/100 participants)	242.42	196.97	311.57	341.11	39.39
**Climate**					
**Total CO2 emissions (kg) from Atmosfair (attributed to country classification of participant locations)**					
Low	8459	7790	16658	16310	0

Lower middle	44,185	36,155	61,287	69,227	0
Upper middle	5,443	3,722	15,432	15,711	0
High	117,575	241,085	219,433	323,396	0
**Total**	**175,662**	**288,752**	**312,810**	**424,644**	**0**
**Total Co2 emissions (metric tons) (attributed to country classification of participant locations)**					
Low	8.5	7.8	16.7	16.3	0
Lower middle	44.2	36.2	61.3	69.2	0
Upper middle	5.4	3.7	15.4	15.7	0
High	117.6	241.1	219.4	323.4	0
**Total**	**176**	**289**	**313**	**425**	**0**
per participant (kg)	591	729	928	1,238	0
High-income (%)	67	83	70	76	0
Per-participant for high-income (kg)	1,755	2,318	1,650	2,432	0
**Participant satisfaction (overall rating of the event by participants (1-5 Likert scale)**					
5 (most favorable)	27 (30.0%)	49 (35.0%)	65 (42.5%)	84 (62.2%)	100 (54.9%)
4	42 (46.7%)	71 (50.7%)	73 (47.7%)	44 (32.6%)	64 (35.2%)
3	16 (17.8%)	14 (10.0%)	14 (9.2%)	7 (5.2%)	15 (8.2%)
2	4 (4.4%)	6 (4.3%)	1 (0.7%)	0 (0.0%)	3 (1.6%)
1 (least favorable)	1 (1.1%)	0 (0.0%)	0 (0.00%)	0 (0.0%)	0 (0.0%)
Total responses	90	140	153	135	182
Response rate (% of total participants)	30.3%	35.4%	45.4%	39.4%	19.9%
**Twitter engagament**					
Total tweets + retweets + replies with official hashtag (all time)	613	1,322	1,822	1,851	1,430
Normalised (p/100 participants)	206	334	541	540	156

Note: [1]Nepal's classification was raised from lower income to lower middle income in fiscal year (FY) 2021. The classification was unchanged across years for all other host countries. [2]The specific rating questions are in Section B of the online supplemental materials. For years 2017-2021, the ratings came from a single overall participant survey question. For year 2016, the survey lacked an overall rating question, so the rating is a participation-weighted average of ratings from session-specific ratings.

B. Participant satisfaction: Conference rating questions

2020

5 Please rate the 2020 ANH Academy Week. (Please select 1 answer)

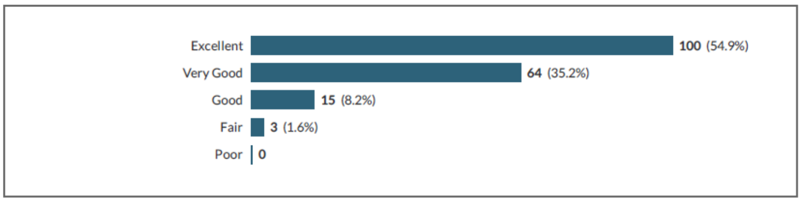

2019

5 Please rate the 2019 ANH Academy Week. (Please select 1 answer)

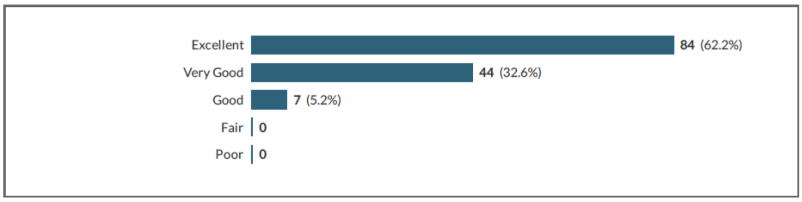

2018

5 Please rate the 2018 ANH Academy Week.

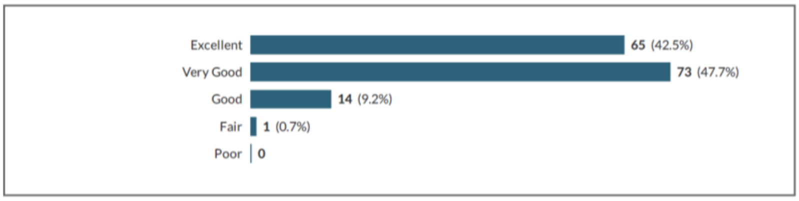

2017

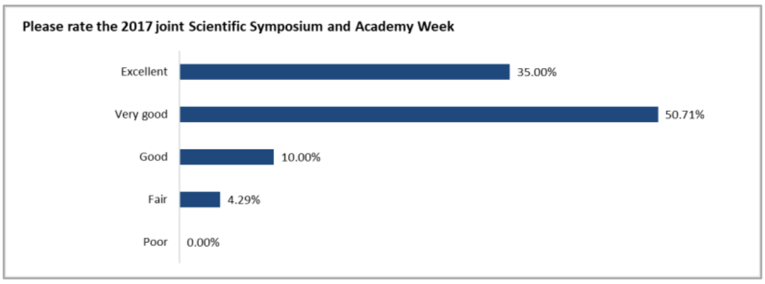

2016*

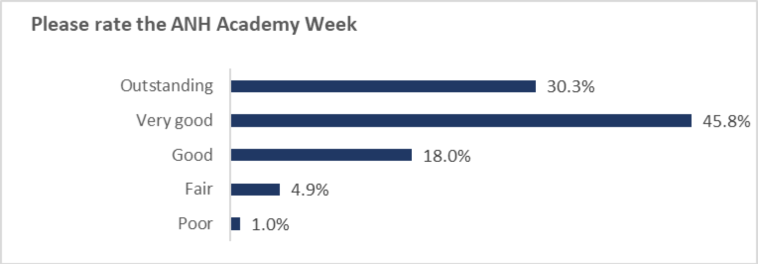

*For 2016, data are aggregated from session-level feedback responses using the same Likert scale
